# A 'small-world-like' model for comparing interventions aimed at preventing and controlling influenza pandemics

**DOI:** 10.1186/1741-7015-4-26

**Published:** 2006-10-23

**Authors:** Fabrice Carrat, Julie Luong, Hervé Lao, Anne-Violaine Sallé, Christian Lajaunie, Hans Wackernagel

**Affiliations:** 1 Université Pierre et Marie Curie-Paris6, INSERM, UMR-S 707, Paris F-75012, France; 2 Assistance Publique Hôpitaux de Paris, Hôpital Saint-Antoine, Paris F-75012, France; 3 Centre de Géostatistique de l'Ecole des Mines de Paris, Fontainebleau F-77300, France

## Abstract

**Background:**

With an influenza pandemic seemingly imminent, we constructed a model simulating the spread of influenza within the community, in order to test the impact of various interventions.

**Methods:**

The model includes an individual level, in which the risk of influenza virus infection and the dynamics of viral shedding are simulated according to age, treatment, and vaccination status; and a community level, in which meetings between individuals are simulated on randomly generated graphs. We used data on real pandemics to calibrate some parameters of the model. The reference scenario assumes no vaccination, no use of antiviral drugs, and no preexisting herd immunity. We explored the impact of interventions such as vaccination, treatment/prophylaxis with neuraminidase inhibitors, quarantine, and closure of schools or workplaces.

**Results:**

In the reference scenario, 57% of realizations lead to an explosive outbreak, lasting a mean of 82 days (standard deviation (SD) 12 days) and affecting 46.8% of the population on average. Interventions aimed at reducing the number of meetings, combined with measures reducing individual transmissibility, would be partly effective: coverage of 70% of affected households, with treatment of the index patient, prophylaxis of household contacts, and confinement to home of all household members, would reduce the probability of an outbreak by 52%, and the remaining outbreaks would be limited to 17% of the population (range 0.8%–25%). Reactive vaccination of 70% of the susceptible population would significantly reduce the frequency, size, and mean duration of outbreaks, but the benefit would depend markedly on the interval between identification of the first case and the beginning of mass vaccination. The epidemic would affect 4% of the population if vaccination started immediately, 17% if there was a 14-day delay, and 36% if there was a 28-day delay. Closing schools when the number of infections in the community exceeded 50 would be very effective, limiting the size of outbreaks to 10% of the population (range 0.9%–22%).

**Conclusion:**

This flexible tool can help to determine the interventions most likely to contain an influenza pandemic. These results support the stockpiling of antiviral drugs and accelerated vaccine development.

## Background

There are increasing concerns that an A/H5N1 influenza pandemic is imminent. Based on data from recent pandemics, 50 countries have developed pandemic preparedness plans and most industrialized countries are stockpiling antiviral drugs [1]. An international workforce has been created to develop an H5N1 vaccine [2], and immunogenicity trials are promising [3,4].

Public health decision-making will be based largely on experience with past pandemics, but models are needed to plan and evaluate interventions based on vaccination, antiviral prophylaxis/therapy, quarantine, and closure of public places. As the transmissibility and pathogenicity of emerging influenza viruses cannot be predicted, and neither can their pandemic potential, such models should be flexible enough to be adapted to a wide range of situations. They must deal with various types of populations and test different kinds of interventions, used together or in isolation.

Recent papers focus on the containment of an outbreak in a rural area of Southeast Asia, where a pandemic virus seems most likely to emerge [5,6], or on strategies for mitigating the severity of a pandemic in the United States or Great Britain, where a virus is likely to spread secondarily [7,8]. The authors used different methodologies, but the results of both studies showed that a nascent pandemic could be contained by using a combination of antiviral drugs and confinement measures. Another paper suggested that, in the United States, vaccination (particularly of children) could be very effective [9].

We have developed a model for simulating the spread of influenza virus infection in the community during a pandemic. The model includes not only individual parameters, which take into account the risk of infection and the dynamics of viral shedding according to age, treatment, and vaccination status, but also community parameters, in which meetings between individuals are simulated by the use of a complex random graph.

## Methods

### Individual-centered model of influenza infection, illness, and health-care use

A computer model was first developed to describe influenza infection and its consequences for a given individual. We used the classical four-stage model of infection, as follows: Susceptible (S – may be infected), Exposed (E – is infected but cannot transmit the disease), Infectious (I – is infected and can transmit the disease), and Recovered (R – can no longer transmit the disease and is immune to new infections).

The three basic parameters used to describe transitions between the different stages were the person-to-person transmission rate, which is assumed to vary with the age of susceptible and infectious individuals and with the time since infection; the length of the latent period (time between infection and onset of infectivity); and the length of the infectious period.

**Figure 1:**
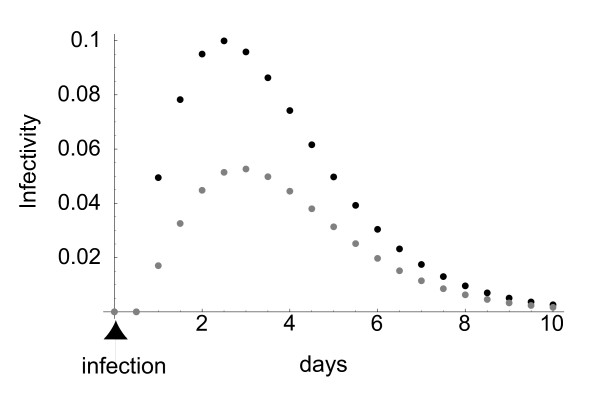
**Infectivity profiles of individuals according to time since influenza infection**. A latent period of 0.5 days was postulated. Black dots represent infectivity in children and grey dots, infectivity in adults or elderly subjects.

In order to obtain a biologically realistic description of the person-to-person transmission process, we assumed that infectivity varies with time since infection and is proportional to the degree of viral shedding by infected individuals (Table 1). Based on data from experimental studies in which viral shedding was measured in volunteers challenged with wild-type influenza viruses [10,11], we modeled the kinetics of infectivity by using [l.c. gamma] density functions with a fixed offset of 0.5 days, corresponding to the latent period (Figure 1). The profiles thus obtained were consistent with those of a prospective household-contacts survey conducted in France, with peak infectivity between the second and third days after infection, infectivity lasting a maximum of 10 days, and 1.8-fold-higher daily infectivity of children compared with adults [12,13]. Finally, we modulated individual susceptibility by age, again based on the results of the prospective survey, in which we showed that susceptibility was higher in children than in adults [14]. The infectivity profiles were then scaled by a factor that was identical for children and adults in order to obtain attack rates consistent with those reported during pandemics in children and adults or the elderly (see below). The resulting probability of transmission during a hypothetical meeting lasting throughout the infective period between a susceptible child and a single infected child was 64%; the corresponding probability of transmission between a susceptible adult and a child was 58%; the corresponding values for permanent meetings between a single infected adult and a susceptible child and adult were 42% and 37%, respectively.

As influenza virus infection is not always symptomatic, we postulated that 30% of infected individuals would not be sufficiently ill to be identifiable [6], and that these subjects would be half as infective as other subjects. For symptomatic individuals, we postulated that the duration and intensity of symptoms would be proportional to infectivity, based on the observation that the onset of symptoms after experimental infection coincides with a sharp increase in viral shedding [10,15-21], i.e. the incubation period is equal to the latent period.

For case and contact tracing, and for access to interventions (treatment, prophylaxis, etc.), patients must be seen by a physician. We postulated that most symptomatic subjects would seek medical advice (90%), and that 40% of those who consulted would do so within the first day after onset, 30% the second day, and 30% after the second day. These rates were chosen to be higher than those observed during a seasonal influenza epidemic [12], as public awareness would be higher in a pandemic situation and as antiviral treatment would be available only from a physician. Finally, we postulated that 80% of individuals who consulted a physician would remain confined to their home for one week.

We postulated that 5% to 13% of symptomatic subjects (depending on age) would be hospitalized for serious complications and that 20% to 30% of those hospitalized would die. The case-fatality rates thus ranged from 1% to 4%, in keeping with data collected during previous pandemics [22,23] The average hospital stay was set at 12 days, based on French national statistics on hospitalization for pneumonia and influenza [24]. We postulated that transmission could not occur between patients or from patients to hospital staff, owing to strict application of preventive measures.

### Community model

The community model was based on a complex random graph realistically describing meetings between individuals. We first generated a set of individuals based on a particular demographic profile (gender, age groups, and household sizes) adapted from French national census data [25], in which each individual is assigned to a household and a place of occupation (for example, a school for a child, or a workplace for a working adult). Households and places of occupation were assigned to districts, and children were preferentially assigned to schools located in the district where they lived; 20% of working adults were assigned to workplaces located in other districts. In the reference simulation, 23% of individuals were children, 67% were adults (80% in employment), and 10% were elderly.

Two types of bidirectional graphs were generated. First, a fully connected graph was generated for each household, as we assumed that every household member would make daily meetings with all other household members (if any).

For schools, workplaces and other locations (nursing homes, hospital, etc), meetings between individuals were modeled with the Barabasi-Albert (BA) random graph [26]. The BA graph was developed in the late 1990s to describe systems in which the probability that a node will have a given number of connections with other nodes does not depend on the size of the system. This type of graph can correctly describe systems such as links on the worldwide web and citations in scientific journals [27,28]. It can also provide a realistic representation of social contacts: the first application of this method was to describe the network of movie actors [28]).

BA graphs are built up from a small initial numbers of nodes (three, for example), in two steps: a growth step, in which a new node with *m *connections is added; and a preferential attachment step, in which the nodes to which the new node connects are chosen. The probability Π that the new node will be connected to node *i *depends on the connectivity *k*_*i *_of that node, such that Π(ki)=ki/∑jkj
 MathType@MTEF@5@5@+=feaafiart1ev1aaatCvAUfKttLearuWrP9MDH5MBPbIqV92AaeXatLxBI9gBaebbnrfifHhDYfgasaacH8akY=wiFfYdH8Gipec8Eeeu0xXdbba9frFj0=OqFfea0dXdd9vqai=hGuQ8kuc9pgc9s8qqaq=dirpe0xb9q8qiLsFr0=vr0=vr0dc8meaabaqaciaacaGaaeqabaqabeGadaaakeaacqqHGoaucqGGOaakcqWGRbWAdaWgaaWcbaGaemyAaKgabeaakiabcMcaPiabg2da9maalyaabaGaem4AaS2aaSbaaSqaaiabdMgaPbqabaaakeaadaaeqbqaaiabdUgaRnaaBaaaleaacqWGQbGAaeqaaaqaaiabdQgaQbqab0GaeyyeIuoaaaaaaa@3D35@. The probability density *P*(*k*) that a node in the network is connected to *k *other nodes is independent of the size of the system and has a power law distribution, that is *P*(*k*) ~ *Ak*^-γ^, where [l.c. gamma] is 3 and coefficient *A *is proportional to the square average connectivity of the network (*A *~ *m*^2^). The average connectivity of a BA graph is 2*m*.

Various BA graphs were generated for the various locations simulated here (Table 2).

**Figure 2:**
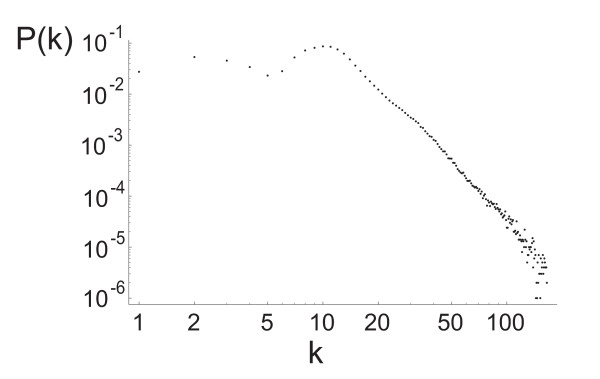
Connectivity distribution of the simulated population.

Figure 2 describes the resulting connectivity (*k*) of the simulated population (100 simulations). The connectivity clearly followed a power-law distribution for *k *values >10. The mean connectivity was 11.9 (standard deviation (SD) 0.28), with differences according to age: 13.6 (SD 0.06) for children, 12.3 (SD 0.41) for adults, and 4.8 (SD 0.14) for elderly people. We also calculated a weighted connectivity by scaling each connection by a factor representing the part of the week during which individuals met and during which transmission could occur if one individual was infectious and the other susceptible. For example, meetings between household members, assumed to occur every morning and every evening of each working day and during the entire weekend, corresponded to 9/14ths of a week. Meetings between employees or school children were equivalent to 5/14ths of a week. The resulting weighted connectivity was 3.87 (SD 0.09), meaning that an individual in our simulated network had an average of nearly 4 permanent meetings with other individuals. We characterized the mixing of the simulated population by computing a mean local clustering coefficient *C*, defined as the mean fraction of existing connections between contacts of each individual. *C *reflects the existence of cliques, or communities: it is the mean probability that two individuals are connected, given that they share a common network contact. The mean local clustering coefficient of the simulated graphs was 0.20 (SD 0.02). Finally, the mean shortest path (the minimum number of contacts) between two randomly chosen individuals in our simulated population was 3.6 (SD 0.15). Thus, our networks exhibited substantial clustering and small-world properties consistent with current knowledge of human social networks [29].

### Simulation process and empirical calibration

Each simulation started with the generation of a network of 10,000 individuals and one infected individual. In order to deal with heterogeneities of susceptibility or connectivity between individuals, we proceeded as follows: we first randomly chose one infected individual and then simulated the first generation of secondary infections. Then each individual infected during the first generation was used as the initial infective in a new simulation where the network and the population were reset to their initial values. The selection of an individual from the first generation ensures proper sampling of the initial infected individual in a heterogenous contact network [30].

A discrete time step (half a day) was chosen. At each time point, meetings between infectious and susceptible individuals were derived from the graph, and transmission of influenza virus during each meeting was simulated by comparing a uniform random number with the calculated probability of transmission. The per-meeting probability of transmission was calculated as the product of infectivity (depending on time since infection) and the relative susceptibility of the contact, and was adjusted for other parameters (vaccination, treatment, etc.). The simulations stopped after the maximal length of the infectious period following the last transmission event.

A critical parameter in the epidemiology of infectious diseases is the basic reproductive number (*R*_0_). *R*_0 _is defined as the average number of secondary infections produced by a single infected person in a fully susceptible population. In our model, analytical calculation of *R*_0 _is not feasible [6]. For this reason, we proceeded by simulation, randomly choosing one infective subject as described above, and then counting the number of secondary infections.

**Figure 3:**
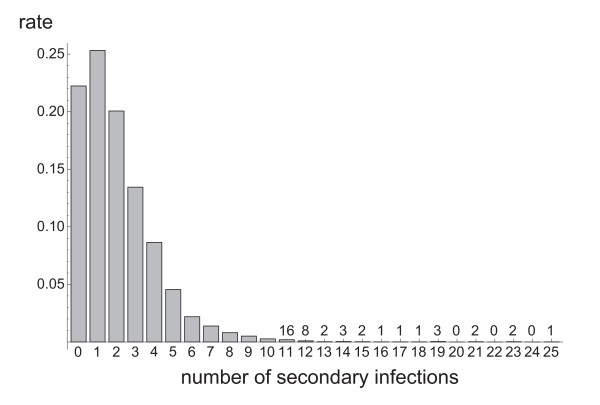
**Distribution of the basic reproductive number *R*_0_**. There were 8000 simulations. Superscripts indicate the numbers of simulations generating a number of secondary infections greater than 10.

Figure 3 shows the distribution of the numbers of secondary infections averaged over 8000 trials. In 22.2% of trials, no secondary cases were generated by the introduction of a single infectious individual into the community. The mean *R*_0 _was 2.07 and the disease generation time, which represents the mean interval between infection of a given person and infection of all the people that this individual infects, was 2.44 (SD 1.48) days.

**Figure 4:**
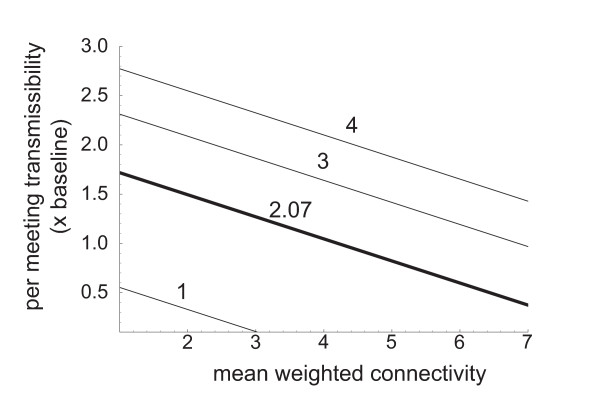
**Sensitivity analysis of the basic reproductive number *R*_0_**. The figure shows the isopleth of *R*_0 _as a function of weighted connectivity and multiples of baseline per-meeting probabilities of transmission. The bold line corresponds to *R*_0 _= 2.07. Curves were plotted using the following regression equation: *R*_0 _= Exp([minus]0.485 + 0.347 [multiplication sign] multiple of the baseline per-meeting probability of transmission + 0.14 weighted connectivity).

We then explored the sensitivity of the basic reproductive number to the number of meetings and to the per-meeting probability of transmission. Parameters describing the meetings (mean weighted connectivity between 1 and 7) and per-meeting transmissibility (0.1 to 3 times the reference value) were varied on a 10 [multiplication sign] 10 grid with 40 simulations for each combination of parameters. Normal regression analysis with a log link was performed with the mean number of secondary cases as the response variable and weighted connectivity and per-meeting transmissibility as predictors. As expected, the mean weighted connectivity and the per-meeting transmissibility correlated independently with the basic reproductive number (Figure 4).

The observed rates of seroconversion and illness due to the pandemic strains that circulated during the 20^th ^century were used to calibrate the model, and particularly to scale infectivity. During the 1957 pandemic, serological infection rates as high as 75% were observed among children and 25% among adults [31]. In 1918, during the first pandemic wave, the attack rate of clinical influenza was maximum in children (40%) and then fell gradually with age, reaching 9% in people aged 75 years or more. An average attack rate of 34% was reported during the 1957 pandemic, with an age distribution similar to that observed during the first pandemic wave of 1918 [23]. The age distribution of attack rates during the 1968 pandemic was noticeably different, with values decreasing less markedly with age, ranging between 41% and 43% in children, but remaining above 30% in all other age groups [23]. Most of these rates were obtained from studies of families with children (which tend to overestimate the true attack rates in the general population), but served as benchmarks for empirical calibration of our model. The shape and length of the pandemic curve were also consistent with those reported in cities during the 1918 pandemic [32].

## Results

### Reference scenario

**Figure 5:**
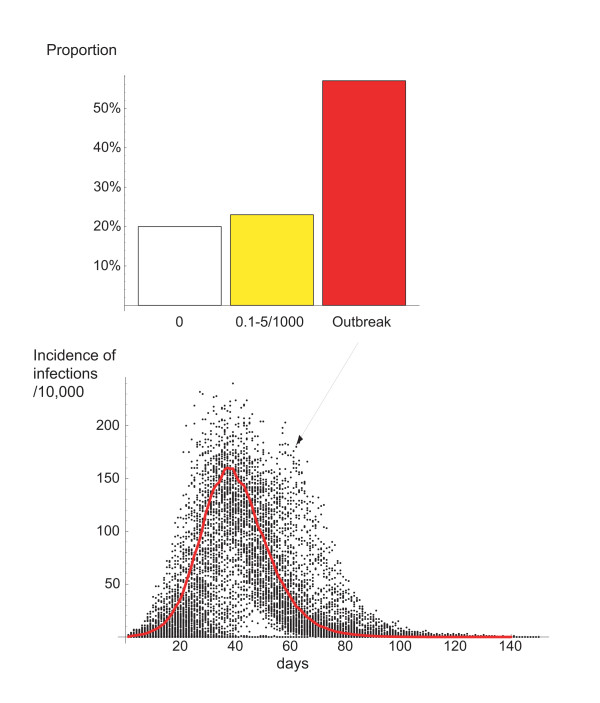
**Simulation of the reference scenario for a flu pandemic (no intervention)**. The top figure describes the distribution of the numbers of secondary cases following introduction of a single infected individual into the population (200 simulations), and the bottom figure describes the infection curves of simulated outbreaks. The bold line is the average of the simulated outbreaks.

Two hundred realizations were simulated for each scenario. Three patterns were observed. No secondary infections were generated in 20% of simulations (see above). In 23% of simulations, a limited number of infections occurred and the epidemics always affected fewer than five subjects per 1000 (Figure 5). In the remaining 57% of simulations, explosive growth occurred and the epidemic affected an average of 46.8% of subjects (SD 1.7%). The mean duration of the outbreaks, defined as the time between the first secondary infection and the last infection, was 82 days (SD 12 days). The cumulative incidence rate of influenza infection was much higher in children than in adults, including the elderly. The mean clinical attack rate was 33% (SD 1%), 1.7% (SD 0.16%) of the population was hospitalized, and 0.36% (SD 0.07%) died from influenza (a value intermediate between the 1918 pandemic (0.6%) and the following two pandemics (0.04%–0.01%) [33]). The number of workdays lost per working adult was 1.37 (SD 0.07). Other results, averaged over 200 realizations, are shown in Table 3.

### Intervention scenarios

We first simulated the effectiveness of neuraminidase inhibitors in individuals who sought medical advice and were treated for five days. We assumed that treatment reduced infectivity and clinical severity (including the risk of complications and death) by 28% [5]. We also assumed that treatment would not affect the mean number of workdays lost per patient. Table 4 shows the results for a treatment coverage rate of 90%. An outbreak was simulated in 53% of cases and the size of the outbreaks and the clinical attack rate were only slightly affected by treatment, owing to a decrease in transmissibility (Table 4). However, the rates of hospitalization and death decreased, mainly as a result of a lower risk of complications in treated individuals. It is noteworthy that drug stockpiles sufficient for 25% of the population coverage would permit the treatment of 90% of patients who consult a physician.

Several randomized controlled trials have demonstrated the preventive effectiveness of neuraminidase inhibitors (see [34] for a recent review). We postulated that a 10-day course of prophylaxis with neuraminidase inhibitors would reduce susceptibility to influenza virus infection by 80% during each meeting [35,36]. We tested two scenarios, one with prophylaxis of household contacts but no treatment of the index case, and one combining treatment of the index case and prophylaxis of household contacts. Table 5 shows the results for 70% coverage of household contacts and index cases. Combined treatment and prophylaxis would slightly reduce the burden of influenza outbreaks by comparison with contact prophylaxis without treatment of index cases.

**Figure 6:**
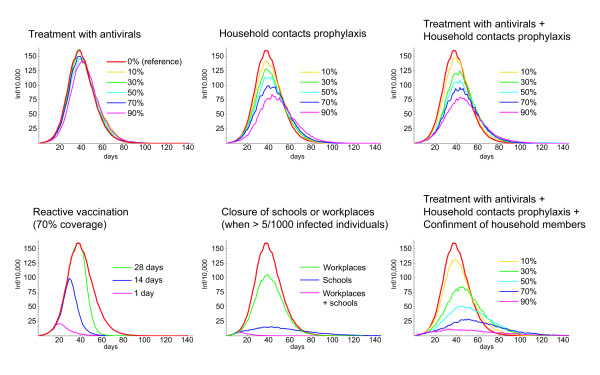
Impact of interventions on infection curves of simulated outbreaks.

We then examined the impact of 10-day confinement to home of all members of households in which a case was identified by a physician, combined with prophylaxis of household contacts and treatment of the index case. This strategy would increase effectiveness by comparison with similar scenarios not involving confinement: coverage of 70% of affected households would be sufficient to reduce the risk of an outbreak by 52%, restricting it to 17% of the population (range 0.8%–25%) (Figure 6). The mean duration of the outbreak would be increased (119 days, SD 22) by comparison with the reference scenario and influenza virus would persist in the population for more than five months in 25% of simulations.

We also modeled a scenario in which mass vaccination would begin a certain time after identification of the first case (0, 14, 28 days) and in which the target level of vaccine coverage would be achieved within 14 days. We postulated that individual protective immunity would be achieved two weeks after vaccination and that vaccination would reduce susceptibility by 80% during each meeting (leaky vaccine, meaning that vaccinated individuals would respond by acquiring partial immunity, rather than acquiring either complete immunity or no immunity at all [37]). Mass vaccination could take place in schools, workplaces, nursing homes, hospitals, and physicians' offices. We assumed that vaccination would lead to the loss of 0.04 workdays per working adult [38].

Reactive mass vaccination would significantly reduce the frequency, size, and mean duration of outbreaks (Figure 6), but the benefit would depend closely on how long it took to begin vaccination after identification of the first case (Table 6).

Finally, we simulated an intervention in which schools and workplaces are closed when a threshold number of infections (5/1000 subjects in our example) has been reached in the population and are reopened 10 days after the last observed case of infection. This strategy could be used if vaccines and/or antiviral drugs were in short supply or ineffective. Table 7 shows the results of closure of schools alone or both schools and workplaces. This strategy would be very effective, but would clearly be associated with massive time off work.

## Discussion

Using a realistic description of influenza infection in the individual subject, we show that an influenza pandemic with a burden comparable to that of 20th-century pandemics might be mitigated by combining measures aimed at reducing meeting frequency and virus transmissibility. This conclusion is based on several assumptions [5,6] and would be influenced by average infectivity, variability of infectivity [39], and the frequency and patterns of meetings between individuals [40], as these two dimensions govern the basic reproductive number. We found that an average *R*_0 _of 2.07 can provide attack rates and pandemic curves consistent with those reported in previous pandemics, including the devastating 1917/1918 pandemic. This value was consistent with that reported in previous studies, where *R*_0 _ranged from 1.4 to 2.4 [7,8]. It should be noted that a random choice of the initial infected individual would lead to a strong underestimation of *R*_0 _(1.4 in our model). Findings would also be most sensitive to parameters governing the natural history of influenza illness or health-care use. One-way sensitivity analysis showed that the lengths of the latent or incubation periods or the proportion of physician visits occurring during the first day of illness might strongly modify the dynamics of the epidemic or the effectiveness of interventions (see Additional files 1 and 2). Changes that occur during epidemics, such as increased virus fitness for human-to-human transmission [41] and spontaneous changes in meeting rates in response to the perceived risk, must also be considered. The severity of an epidemic would also be highly sensitive to the efficacy of preventive or therapeutic treatments or vaccination, efficiency of case identification, timely implementation of control measures, population coverage, and public compliance [42]. The number of unknown parameters is too large for meaningful sensitivity analysis. In addition, the characteristics of the next pandemic influenza virus strain cannot be reliably predicted, and neither can the effectiveness of control measures. For example, a critical factor not included in this work is the possible emergence of resistance to antiviral drugs [43,44]. However, it could be useful to collect pandemic-independent information on patterns of social meetings and the precise mechanism by which influenza usually spreads during winter epidemics in temperate countries. The choice of the BA scale-free network for describing person-to-person meetings within places of occupation may be questionable [45], but BA networks generate broad heterogeneity in meeting patterns, which may contribute to generating 'superspreading' events [46]. The 20/80 rule, which suggests that 20% of individuals are responsible for 80% of transmission events, can be tested on epidemiological datasets [47].

## Conclusion

This flexible tool can help to determine the interventions most likely to contain an influenza pandemic. At present, our results support the stockpiling of antiviral drugs and accelerated development of vaccines.

## Abbreviations

BA Barabasi-Albert

SD standard deviation

## Competing interests

F Carrat has received fees for consultancies from Roche, GlaxoSmithKline, Chiron, and Aventis in the past five years. The other authors declare that they have no competing interests.

## Authors' contributions

FC conceived and supervised the study and drafted the manuscript. JL, HL, and VS developed the simulation model and carried out the simulations. CL and HW conceived the study and helped draft the manuscript. All authors read and approved the final manuscript.

## Pre-publication history

The pre-publication history for this paper can be accessed here:



## Supplementary Material

Additional File 1 **PDF file One-way sensitivity analysis figures**. A PDF file showing one-way sensitivity analyses of the reference scenario (Figure 7a) and a "combined interventions" scenario including the coverage of 70% of affected households, with treatment of the index patient, prophylaxis of household contacts, and confinement to home of all household members (Figure 7b), to parameters governing the natural history of influenza infection or healthcare use. The red curves describe simulated outbreaks with the parameter values used in the manuscript. Click here for file

Additional File 2 **Word file One-way sensitivity analysis table**. A Word file showing one-way sensitivity analyses of the reference and the "combined interventions" scenarios to parameters governing the natural history of influenza infection or healthcare use. Click here for file

## Figures and Tables

**Table 1:** Parameters describing the transmissibility and pathogenicity of influenza virus.

Parameter	Baseline values	Sources
*Infectivity profiles*		Adapted from [13], and consistent (to a scale factor) with [10,15–21]
Latent period	0.5 days	
Peak	2.5–3 days	
Duration	<10 days	
*Relative susceptibility*		[5,13]
Children (0–18 years)	1.15	
Adults (19–65 years)	1	
Elderly (>65 years)	1	
*Proportion of asymptomatic individuals (children, adults, elderly people)*	30%	[48]; also used in [6]
*Relative infectivity of asymptomatic individuals*	50%	Assumption also used in [6]

**Table 2:** Parameters describing the community model simulating the spread of influenza.

Place	Size	Graph	Assignment	Meetings
Households	1 to 6	Fully connected		D
Schools				
Elementary	5 classes; 20 children and 2 adults per class	Each class is modeled using a BA graph (*m *= 2); supplementary random links between individuals belonging to different classes.	Children living in the district	WD
Secondary	13 to 15 classes; 30 students and 3 teachers per class	Children and students are linked to teachers.	One college for 5 districts	WD
Workplaces	6 to 3000 according to Zipf distribution [49]	BA graph (*m *= 6)	80% from the district; 20% from outside the district	WD
Nursing homes	45 elderly people, 50 employees per nursing home	BA graph (*m *= 6)		WD
District	All individuals	BA graph (*m *= 1)		WE

BA Barabasi-Albert; D every day; WD every working day; WE every weekend

**Table 3:** Reference scenario for a flu pandemic after one initial case (no intervention). Estimates are cumulative numbers per 100 inhabitants, unless otherwise specified.

Outcomes	Outbreak *n *= 114		All simulations *n *= 200
	
	Mean	Minimum-Maximum	Mean
Infections			
*Total*	*46.8*	*42.3–50.5*	*26.7*
Children (0–18 years)^*a*^	76.5	71.9–79.7	43.6
Adults (19–65 years) ^*a*^	39.9	34.8–44.0	22.8
Elderly (>65 years) ^*a*^	25.3	20.8–30.1	14.4
Physician visits	31.2	28.0–33.7	17.8
Hospital admissions	1.74	1.30–2.30	0.99
Deaths	0.36	0.17–0.55	0.21
Lost workdays^*b*^	137	118–150	78

^*a*^Per 100 individuals of a given age

^*b*^Per 100 working adults

**Table 4:** Treatment with neuraminidase inhibitors of 90% of individuals consulting a physician for 'flu-like' symptoms. Estimates are cumulative numbers per 100 inhabitants, unless otherwise specified.

Outcomes	Outbreak; *n *= 106		All simulations; *n *= 200
	
	Mean	Minimum-Maximum	Mean
Infections			
*Total*	*43.3*	*38.5–48.0*	*23.0*
Children^*a*^	72.5	67.9–76.4	38.4
Adults^*a*^	36.5	30.6–41.8	19.3
Elderly^*a*^	22.3	18.6–26.0	11.8
Physician visits	28.0	24.3–31.2	14.9
Hospital admissions	0.98	0.66–1.18	0.52
Deaths	0.21	0.12 – 0.32	0.11
Lost workdays^*b*^	125	107–141	66
Treatment units (doses)^*c*^	243	215–269	129

^*a*^Per 100 individuals of a given age

^*b*^Per 100 working adults

^*c*^Two per day of treatment, five days per patient

**Table 5:** Household contact prophylaxis with antiviral drugs, with or without treatment of the index cases. The interventions are applied in 70% of households in which one member consults a physician. Estimates are cumulative numbers per 100 inhabitants, unless otherwise specified.

Outcomes	Prophylaxis (Outbreak, *n *= 90)		Prophylaxis + treatment (Outbreak, *n *= 98)	
	
	Mean	Minimum-Maximum	Mean	Minimum-Maximum
Infections				
*Total*	*36.1*	*32.1–40.1*	*34.2*	*30.8–37.4*
Children^*a*^	61.8	57.0–66.2	58.5	53.1–64.5
Adults^*a*^	30.2	25.6–35.0	28.8	24.2–33.4
Elderly^*a*^	16.8	11.2–21.2	14.9	11.3–18.9
Physician visits	23.8	21.8–27.0	22.2	19.6–24.8
Hospital admissions	1.18	0.70–1.57	0.77	0.59–0.98
Deaths	0.25	0.13–0.43	0.16	0.06–0.28
Lost workdays^*b*^	106	94–122	100	85–116
Treatment units (doses)^*c*^	196	179–210	393	356–435

^*a*^Per 100 individuals of a given age

^*b*^Per 100 working adults

^*c*^Two per day of treatment, five days per patient; one per day of prophylaxis, 10 days per person

**Table 6:** Reactive vaccination of 70% of the susceptible population according to the interval between implementation and identification of the first case in the community. Estimates are cumulative numbers per 100 inhabitants, unless otherwise specified.

Outcomes	Interval (days)			
	
	0 (Outbreak, *n *= 121)		28 (Outbreak, *n *= 122)	
	Mean	Minimum-Maximum	Mean	Minimum-Maximum

Infections				
*Total*	*4.2*	*0.5–15.7*	*36.1*	*2.0–46.9*
Children^*a*^	8.6	0.7–31.5	62.6	4.6–77.7
Adults^*a*^	3.0	0.4–11.9	29.7	1.4–40.2
Elderly^*a*^	1.6	0.0–5.8	18.1	0.5–27.8
Physician visits^*b*^	1.6	0.0–10.3	23.9	1.3–31.2
Hospital admissions	0.15	0.01–0.5	1.34	0.06–1.92
Deaths	0.033	0–0.14	0.29	0.02–0.51
Lost workdays^*c*^	11	1–44	103	5–137
Vaccination	69.8	69–71	61.6	44–71

^*a*^Per 100 individuals of a given age

^*b*^Physician visits for 'flu-like' illness (excludes visits for influenza vaccination)

^*c*^Per 100 working adults

**Table 7:** Impact of closing institutions when >5 infections per 1000 subjects are observed in the community. Estimates are cumulative numbers per 100 inhabitants, unless otherwise specified.

Outcomes	Closing schools (Outbreak, *n *= 111)		Closing schools and workplaces (Outbreak, *n *= 101)	
	
	Mean	Minimum-Maximum	Mean	Minimum-Maximum
Infections				
*Total*	*9.7*	*0.5–19.5*	*1.1*	*0.6–2.1*
Children^*a*^	10.3	1.6–23.1	2.0	0.6–3.8
Adults^*a*^	9.9	0.6–23.7	0.8	0.4–1.5
Elderly^*a*^	6.1	0.3–14.5	0.8	0.1–3.2
Physician visits	6.4	0.6–14.1	0.7	0.4–1.4
Hospital admissions	0.36	0.03–0.9	0.04	0.0–0.11
Deaths	0.081	0–0.3	0.009	0.0 – 0.04
Lost workdays^*b*^	324	80–464	1885	977–5484
Duration of closure (days)	101	13–107	27	14–78

^*a*^Per 100 individuals of a given age

^*b*^Per 100 working adults
