# Supplementary material for: A 'small-world-like' model for comparing interventions aimed at preventing and controlling influenza pandemics
Source: BMC Med. 2006 Oct 23;4:26. doi: 10.1186/1741-7015-4-26 (PMC1626479; doi:10.1186/1741-7015-4-26)
Supplement: Additional File 1 — PDF file One-way sensitivity analysis figures. A PDF file showing one-way sensitivity analyses of the reference scenario (Figure 7a) and a "combined interventions" scenario including the coverage of 70% of affected households, with treatment of the index patient, prophylaxis of household contacts, and confinement to home of all household members (Figure 7b), to parameters governing the natural history of influenza infection or healthcare use. The red curves describe simulated outbreaks with the parameter values used in the manuscript. [file 1741-7015-4-26-S1.pdf]

Additional file 1.

Figure 7a.

One-way sensitivity analysis of the reference scenario. The red curves describe simulated outbreaks with the parameter values used in the manuscript.

Figure 7b

One-way sensitivity analysis of a “combined interventions” scenario including the coverage of 70% of affected households, with treatment of the index patient, prophylaxis of household contacts, and confinement to home of all household members. The red curves describe simulated outbreaks with the parameter values used in the manuscript.

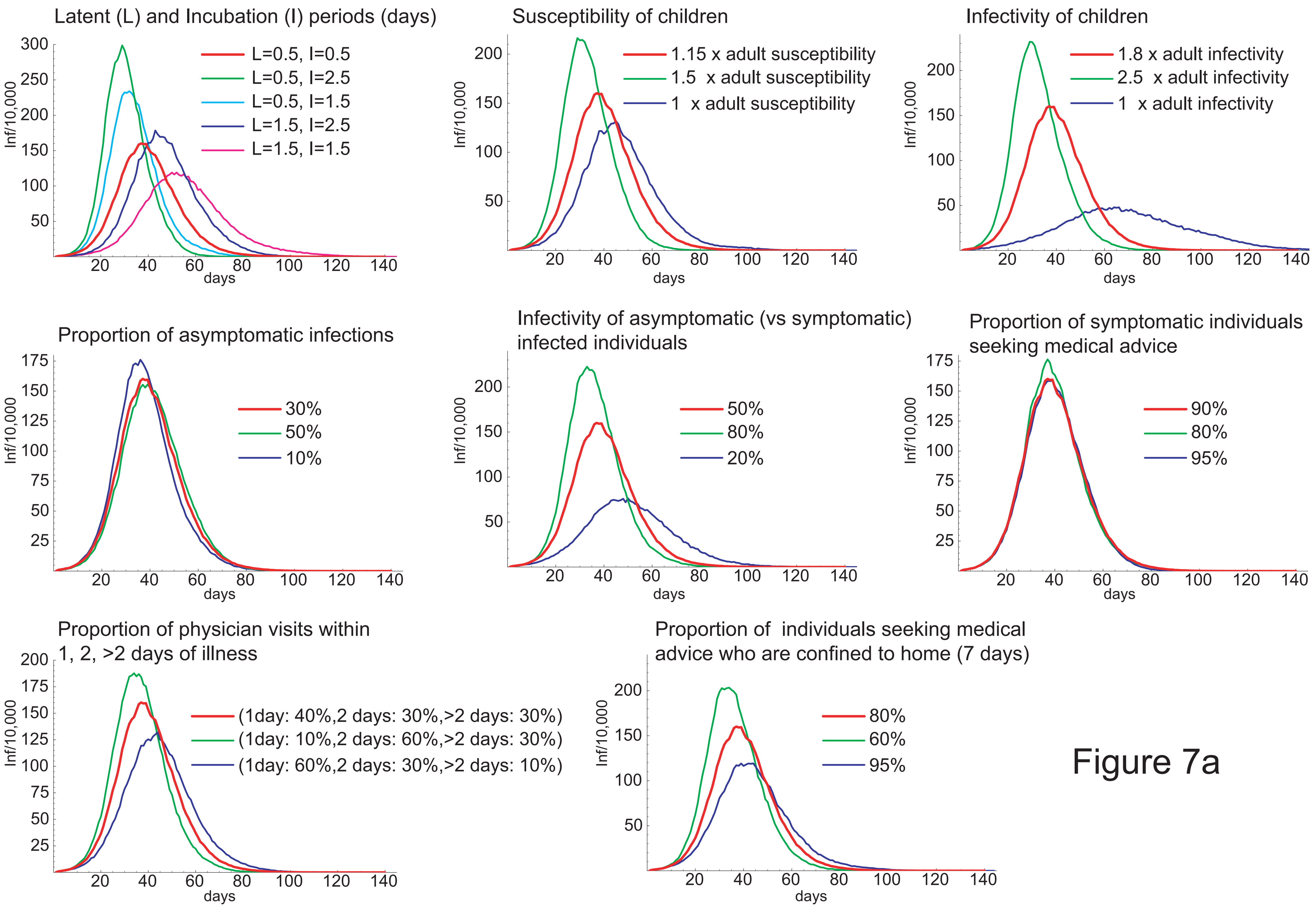

Latent (L) and Incubation (I) periods (days)
L=0.5, I=0.5
L=0.5, I=2.5
L=0.5, I=1.5
L=1.5, I=2.5
L=1.5, I=1.5
Susceptibility of children
1.15 x adult susceptibility
1.5 x adult susceptibility
1 x adult susceptibility
Infectivity of children
1.8 x adult infectivity
2.5 x adult infectivity
1 x adult infectivity
Proportion of asymptomatic infections
30%
50%
10%
Infectivity of asymptomatic (vs symptomatic) infected individuals
50%
80%
20%
Proportion of symptomatic individuals seeking medical advice
90%
80%
95%
Proportion of physician visits within 1, 2, >2 days of illness
(1day: 40%,2 days: 30%,>2 days: 30%)
(1day: 10%,2 days: 60%,>2 days: 30%)
(1day: 60%,2 days: 30%,>2 days: 10%)
Proportion of individuals seeking medical advice who are confined to home (7 days)
80%
60%
95%
Inf/10,000
days

Figure 7a

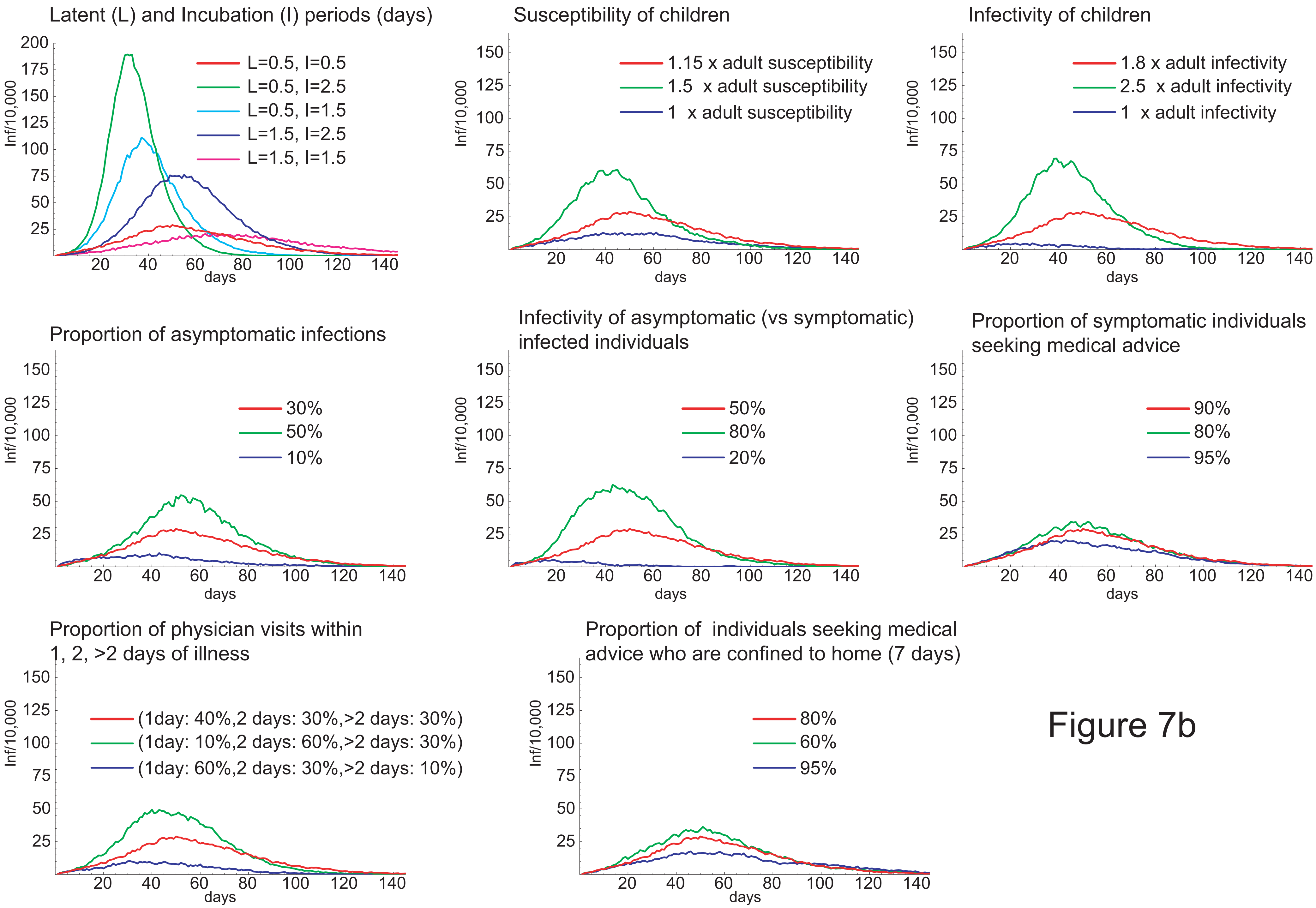

Latent (L) and Incubation (I) periods (days)
L=0.5, I=0.5
L=0.5, I=2.5
L=0.5, I=1.5
L=1.5, I=2.5
L=1.5, I=1.5
Susceptibility of children
1.15 x adult susceptibility
1.5 x adult susceptibility
1 x adult susceptibility
Infectivity of children
1.8 x adult infectivity
2.5 x adult infectivity
1 x adult infectivity
Proportion of asymptomatic infections
30%
50%
10%
Infectivity of asymptomatic (vs symptomatic) infected individuals
50%
80%
20%
Proportion of symptomatic individuals seeking medical advice
90%
80%
95%
Proportion of physician visits within 1, 2, >2 days of illness
(1day: 40%,2 days: 30%,>2 days: 30%)
(1day: 10%,2 days: 60%,>2 days: 30%)
(1day: 60%,2 days: 30%,>2 days: 10%)
Proportion of individuals seeking medical advice who are confined to home (7 days)
80%
60%
95%
Inf/10,000
days

Figure 7b
