# Supplementary material for: A 'small-world-like' model for comparing interventions aimed at preventing and controlling influenza pandemics
Source: BMC Med. 2006 Oct 23;4:26. doi: 10.1186/1741-7015-4-26 (PMC1626479; doi:10.1186/1741-7015-4-26)
Supplement: Additional File 2 — Word file One-way sensitivity analysis table. A Word file showing one-way sensitivity analyses of the reference and the "combined interventions" scenarios to parameters governing the natural history of influenza infection or healthcare use. [file 1741-7015-4-26-S2.doc]

Additional File 2

One-way sensitivity analysis of parameters governing the natural history of influenza illness or healthcare use

|  | Reference scenario | | | | | |  | Treatment of the index patient, prophylaxis of household contacts, and confinement to home of all household members in 70% of affected households | | | | | |
| --- | --- | --- | --- | --- | --- | --- | --- | --- | --- | --- | --- | --- | --- |
|  | Outbreaks  (>5/1000) | Cumulative  Infection rate (%) | | | Duration (days) | |  | Outbreaks  (>5/1000) | Cumulative  Infection rate (%) | | | Duration (days) | |
|  | n | Mean | min | max | Mean | SD |  | n | Mean | min | max | Mean | SD |
| Reference | 114 | 46.8 | 42.3 | 50.5 | 83 | 12 |  | 59 | 16.7 | 0.75 | 24.7 | 119 | 22 |
| Latent = 0.5 days, Incubation = 1.5 days | 139 | 54.9 | 51.6 | 57.8 | 71 | 10 |  | 104 | 35 | 29.7 | 39.2 | 90 | 14 |
| Latent = 0.5 days, Incubation = 2.5 days | 148 | 60.3 | 56.2 | 63 | 64 | 8 |  | 134 | 45.7 | 42.4 | 50.2 | 75 | 10 |
| Latent = 1.5 days, Incubation = 1.5 days | 112 | 46.9 | 41.6 | 51.7 | 108 | 14 |  | 60 | 16 | 0.6 | 26.6 | 130 | 30 |
| Latent = 1.5 days, Incubation = 2.5 days | 122 | 54.6 | 51.6 | 58.4 | 92 | 11 |  | 105 | 35.8 | 30.5 | 40 | 121 | 16 |
| Relative susceptibility of children (vs adults) = 1 | 101 | 43.8 | 41.1 | 48.4 | 94 | 14 |  | 42 | 8.4 | 0.6 | 19.1 | 92 | 38 |
| Relative susceptibility of children (vs adults) = 1.5 | 134 | 50.9 | 47.1 | 54.2 | 70 | 8 |  | 58 | 25.6 | 0.6 | 31.5 | 101 | 20 |
| Relative infectivity of children (vs adults) = 1 | 69 | 29.1 | 1.7 | 34.1 | 134 | 13 |  | 19 | 2.1 | 0.5 | 6.2 | 54 | 32 |
| Relative infectivity of children (vs adults) = 2.5 | 124 | 53.4 | 50.3 | 56.3 | 69 | 10 |  | 71 | 27.2 | 0.8 | 31.9 | 97 | 20 |
| Proportion of asymptomatic infected individuals = 10% | 120 | 47.1 | 41.5 | 50.5 | 81 | 10 |  | 31 | 5.3 | 0.7 | 15.2 | 70 | 34 |
| Proportion of asymptomatic infected individuals = 50% | 117 | 46.6 | 43.7 | 49.9 | 85 | 11 |  | 68 | 25.7 | 0.8 | 31.1 | 120 | 20 |
| Relative infectivity of asymptomatic individuals = 20% | 72 | 31.8 | 0.6 | 37.4 | 103 | 17 |  | 18 | 1.8 | 0.5 | 6.1 | 49 | 24 |
| Relative infectivity of asymptomatic individuals = 80% | 123 | 55.9 | 52.8 | 58.9 | 74 | 9 |  | 84 | 30.2 | 1.1 | 35.7 | 107 | 19 |
| Proportion of symptomatic individuals who seek medical advice = 80% | 116 | 47.6 | 44.3 | 50.5 | 82 | 10 |  | 51 | 18.3 | 0.5 | 28.5 | 103 | 38 |
| Proportion of symptomatic individuals who seek medical advice = 95% | 111 | 46.1 | 0.7 | 50.2 | 81 | 10 |  | 46 | 13.1 | 0.6 | 22.2 | 102 | 36 |
| Proportion of physician visits within 1,2, and >2 days=(10%,60%,30%) | 125 | 49.6 | 45.8 | 53.1 | 76 | 11 |  | 68 | 24.6 | 0.6 | 31.2 | 109 | 19 |
| Proportion of physician visits within 1,2, and >2 days=(60%,30%,10%) | 112 | 43.2 | 38.3 | 47.2 | 92 | 14 |  | 24 | 5.1 | 0.5 | 17.4 | 66 | 32 |
| Proportion of individuals seeking medical advice who are confined to home = 60% | 123 | 53.4 | 50.2 | 56.8 | 75 | 9 |  | 51 | 19.7 | 0.7 | 25.4 | 112 | 28 |
| Proportion of individuals seeking medical advice who are confined to home = 95% | 96 | 40.8 | 37.5 | 45.6 | 91 | 13 |  | 49 | 12.7 | 0.5 | 21.4 | 112 | 37 |
